# Soluble urokinase plasminogen activator receptor (suPAR) predicts critical illness and kidney failure in patients admitted to the intensive care unit

**DOI:** 10.1038/s41598-021-96352-1

**Published:** 2021-09-01

**Authors:** Alexander C. Reisinger, Tobias Niedrist, Florian Posch, Stefan Hatzl, Gerald Hackl, Juergen Prattes, Gernot Schilcher, Anna-Maria Meißl, Reinhard B. Raggam, Markus Herrmann, Philipp Eller

**Affiliations:** 1 Department of Internal Medicine, Intensive Care Unit, Medical University of Graz, Auenbruggerplatz 15, 8036 Graz, Austria; 2 Clinical Institute of Medical and Chemical Laboratory Diagnostics, Medical University of Graz, Graz, Austria; 3 Department of Internal Medicine, Division of Oncology, Medical University of Graz, Graz, Austria; 4 Department of Internal Medicine, Division of Hematology, Medical University of Graz, Graz, Austria; 5 Department of Internal Medicine, Section of Infectious Diseases and Tropical Medicine, Medical University of Graz, Graz, Austria; 6 Department of Internal Medicine, Division of Nephrology, Medical University of Graz, Graz, Austria; 7 Department of Internal Medicine, Division of Angiology, Medical University of Graz, Graz, Austria

**Keywords:** Infectious diseases, Diagnosis, Prognostic markers

## Abstract

Soluble urokinase plasminogen activator receptor (suPAR) is an inflammatory biomarker and risk factor for kidney diseases, with a potential prognostic value in critically ill patients. In this monocentric prospective study, we measured plasma suPAR levels immediately after ICU admission in unselected 237 consecutive patients using a turbidimetric assay. Primary objective was the prognostic value for ICU- and 28-day mortality. Secondary objectives were association with sequential organ failure assessment (SOFA) score, coagulation and inflammation markers, AKI-3 and differences in prespecified subgroups. Median suPAR levels were 8.0 ng/mL [25th-75th percentile 4.3–14.4], with lower levels in ICU survivors than non-survivors (6.7 vs. 11.6 ng/mL, p < 0.001). SuPAR levels were higher in COVID-19, kidney disease, moderate-to-severe liver disease, and sepsis. ICU mortality increased by an odds ratio (OR) of 4.7 in patients with the highest compared to lowest quartile suPAR. Kaplan–Meier overall survival estimates at 3 months were 63% and 49%, in patients with suPAR below/above 12 ng/mL (log-rank p = 0.027). Due to an observed interaction between SOFA score and suPAR, we performed a random forest method identifying cutoffs. ICU mortality was 53%, 17% and 2% in patients with a SOFA score > 7, SOFA ≤ 7 & suPAR > 8 ng/mL, and SOFA score ≤ 7 & suPAR ≤ 8 ng/mL, respectively. suPAR was a significant predictor for AKI-3 occurrence (OR per doubling 1.89, 95% CI: 1.20–2.98; p = 0.006). suPAR levels at ICU admission may offer additional value for risk stratification especially in ICU patients with moderate organ dysfunction as reflected by a SOFA score ≤ 7.

## Introduction

The collective of patients admitted to the intensive care unit (ICU) is fairly heterogeneous. Sepsis, acute kidney injury (AKI) or respiratory failure account for a large proportion of patients, and many patients have pre-existing medical conditions^1–4^. Soluble urokinase plasminogen activator receptor (suPAR) is the circulating form of a membrane-bound protein, named uPAR, which can be found on endothelial cells and immune cells such as neutrophils, activated T-cells and macrophages^5,6^. Usually, suPAR levels are low but increase upon inflammatory stimulation during immune system activation. Increased levels of suPAR emerge in numerous conditions as surrogate of inflammation and can therefore serve as a prognostic marker^7–11^. Higher levels were shown to be associated with worse outcomes in various diseases, and suPAR was able to predict mortality in patients with bacteremia and systemic inflammatory response syndrome^12–15^. Previous studies show that elevated levels of suPAR predict AKI and CKD, for which it was shown to be a risk factor^16,17^. Furthermore, AKI can be mitigated using monoclonal antibodies against uPAR^17^. Nevertheless, the prognostic role of suPAR in patients in an ICU real-world setting has yet to be determined as earlier studies retrospectively assessed the values of suPAR at the end of the study period in selected patient cohorts. Additionally, previous published studies used enzyme-linked immunosorbent assays (ELISA) to determine suPAR levels, which are costly, labor-intensive, and slow. To solve these issues, an automated turbidimetric immunoassay was introduced recently. This method not only enables high throughput testing in routine laboratories but also comes along with significantly reduced time to results at approximately one hour. In this prospective cohort study, we aimed to delineate the role and feasibility of plasma suPAR assessed by an immunoturbidimetric test as a prognostic marker and risk stratification tool in an unselected cohort of consecutive patients admitted to a medical ICU.

## Methods

### Study population and study design

We prospectively recruited all consecutive patients older than 18 years admitted to the ICU of the Department of Internal Medicine at the Medical University of Graz, Austria from July until November 2020. Exclusion criteria were a therapy objective order (“comfort terminal care”) given directly at the time of admission, or a decline to participate. The study protocol was approved by the Institutional Review Board (IRB, Ethics committee Graz) of the Medical University of Graz, Austria (32–472 ex 19/20), registered in the WHO approved German study registry (DRKS00022458) and complied with the Declaration of Helsinki. Written informed consent was obtained from all conscious participants. In comatose non-surviving patients, the local IRB acted as legal guardian and gave consent for participation within the study.

### Definitions and pre-specified subgroups

Subgroups as predefined by the study protocol were patients with active hematological malignant disease, active cancer (non-hematological), moderate to severe liver disease, CKD 3–5, pre-existing chronic obstructive pulmonary disease (COPD), acute myocardial infarction, elective valve surgery or percutaneous intervention, and continuous RRT. Sepsis was defined according to sepsis-3 criteria with a suspected infection by the treating physician and a sequential organ failure assessment (SOFA) score increase by ≥ 2 points^18^. Baseline SOFA score was deduced from previous medical records. Septic shock was specified as vasopressor therapy to maintain a mean arterial pressure (MAP) ≥ 65 mmHg in the absence of hypovolemia, and a lactate level > 2 mmol/L^18^. Acute kidney injury 3 (AKI-3) was defined as by KDIGO guidelines including a creatinine level ≥ 4 mg/dL, an increase of creatinine levels by three times from baseline, urine output ≤ 0.3 ml/kg/h for ≥ 24 h OR anuria for ≥ 12 h, or need for renal replacement therapy (RRT)^19^. AKI-3 was assessed separately on admission and during the ICU stay. Chronic kidney disease (CKD) definition was based on previous creatinine levels available from electronic medical records. Estimated glomerular filtration rate (eGFR) was calculated using the CKD-EPI formula^20^.

### suPAR measurement

We determined suPAR levels in plasma samples anticoagulated with lithium heparin using a turbidimetric immunoassay (suPARnostic® TurbiLatex, ViroGates A/S, Copenhagen, Denmark). The test was applied and used according to the manufacturer’s instructions on a Cobas® 8000 c502 analyzer (Roche Diagnostics, Mannheim, Germany). The lower limit of quantification is 1.8 ng/mL, and the upper limit of quantification is 16 ng/mL. Dilution with human serum albumin 5% solution (1:1 ratio, Baxter) was performed in case levels exceeded the upper limit of detection and quantification. For other laboratory measurements see supplementary data.

### Objectives

The primary objective was to assess the prognostic value of baseline plasma suPAR at ICU admission with regard to ICU- and 28-day mortality. Secondary objectives as prespecified by the study protocol included the association of suPAR with coagulation and inflammatory markers, as well as SOFA score. Furthermore, suPAR-level differences in prespecified subgroups and the diagnostic value of suPAR with regards to AKI-3 were investigated.

### Statistical analyses

All statistical analyses were performed with SPSS 26 (SPSS Inc, Chicago, Illinois, USA) and Stata 15.0 (Stata Corp., Houston, TX, USA). Continuous variables were summarized as medians [25th-75th percentile], and categorical variables as absolute frequencies (%). Associations between variables were computed with cross-tabulations, Mann–Whitney-U-tests, χ^2^-tests, and Fisher’s exact tests, as appropriate. Spearman’s rank-based correlation coefficient was used for correlations analyses. The prognostic associations between 28-day / ICU mortality and other potential baseline predictors were computed with univariable and multivariable logistic regression. Formal adjustment for multiple testing was not performed. Significance level was determined at 0.05.

### Ethics approval and consent to participate

The study protocol was approved by the Institutional Review Board (IRB) of the Medical University of Graz, Austria (32–472 ex 19/20), registered in the WHO approved German study registry (DRKS00022458) and complied with the Declaration of Helsinki. Written informed consent was obtained from all conscious participants. In comatose non-surviving patients, the local IRB acted as legal guardian and gave consent for participation within the study.

## Results

### Baseline characteristics and laboratory results of the study population

We screened 242 patients, of whom 237 were prospectively enrolled (Supplementary Fig. 1). Forty percent of the cohort were female and the median age at enrollment was 65 years [25th-75th percentile: 55–74] (Table 1). The median SOFA score was 7 points^3–10^. The median duration of ICU stay was three days, and the overall ICU and 28-day mortality rates were 27% (63 patients), and 38% (90 patients), respectively.

**Table 1:** Baseline characteristics and laboratory values.

	N (%miss.)	Total cohort (n = 237)	ICU survivor (N = 174)	ICU non-survivor (N = 63)	p
**Baseline variables**					
Age	237 (0%)	65 [55–74]	64 [54–74]	69 [56–75]	0.400
Female sex	237 (0%)	95 (40%)	72 (41%)	23 (37%)	0.499
BMI (kg/m^2^)	237 (0%)	26 [23–29]	26 [23–30]	26 [23–28]	0.538
SOFA score	237 (0%)	7 [3–10]	5 [3–8]	11 [8–13]	< 0.0001
AKI 3 on admission#	237 (0%)	52 (22%)	29 (17%)	23 (37%)	0.001
Sepsis on admission*	237 (0%)	117 (49%)	75 (43%)	42 (67%)	0.001
Septic shock on admission*	237 (0%)	39 (16%)	18 (10%)	21 (33%)	< 0.0001
suPAR levels (ng/mL)	237 (0%)	8.0 [4.3–14.4]	6.7 [3.9–14.0]	11.6 [7.1–19.4]	0.0003
Length of ICU stay (days)	237 (0%)	3 [2–7]	3 [2–6]	5 [2–9]	0.142
**Selected comorbidities**					
CKD G3-5	237 (0%)	66 (28%)	46 (26%)	20 (32%)	0.421
COPD	237 (0%)	37 (16%)	26 (15%)	11 (17%)	0.637
Diabetes	237 (0%)	65 (27%)	42 (24%)	23 (37%)	0.059
Moderate-severe liver disease	237 (0%)	24 (10%)	14 (8%)	10 (16%)	0.078
**Laboratory parameters**					
White blood count (G/L)	237 (0%)	11.0 [6.7–15.0]	10.4 [6.3–14.6]	11.2 [7.9–18.0]	0.066
Hemoglobin (g/dL)	237 (0%)	10.8 [9.2–13.1]	11.1 [9.3–13.2]	10.5 [8.7–12.8]	0.343
Platelets (G/L)	237 (0%)	196 [130–274]	203 [129–274]	179 [130–281]	0.961
Creatinine (mg/dL)	237 (0%)	1.3 [0.9–2.6]	1.1 [0.8–2.4]	1.9 [1.3–3.1]	0.0001
Bilirubin (mg/dL)	234 (1%)	0.6 [0.4–1.2]	0.6 [0.3–1.0]	0.7 [0.4–1.7]	0.025
Albumin (g/dL)	220 (7%)	3.4 [2.8–3.8]	3.4 [2.9–3.9]	3.1 [2.4–3.5]	0.002
Lactate (mmol/L)	229 (4%)	1.3 [0.9–2.6]	1.2 [0.8–2.0]	2.0 [1.2–4.6]	< 0.0001
C-reactive protein (mg/L)	237 (0%)	39 [7–108]	34 [7–101]	53 [9–134]	0.318
Procalcitonin (ng/mL)	225 (5%)	0.28 [0.10–1.25]	0.21 [0.08–1.12]	0.55 [0.20–2.35]	0.002
Interleukin-6 (pg/mL)	217 (8%)	63 [20–291]	36 [13–213]	183 [53–975]	< 0.0001
Total cholesterol (mg/dL)	200 (16%)	120 [92–157]	128 [98–158]	98 [85–155]	0.017
HDL cholesterol (mg/dL)	200 (16%)	34 [22–45]	35 [24–46]	28 [16–38]	0.006
Triglycerides (mg/dL)	202 (15%)	120 [87–173]	120 [86–170]	116 [87–173]	0.942
INR (-)	230 (3%)	1.1 [1.0–1.3]	1.0 [0.9–1.3]	1.1 [1.0–1.6]	0.002
aPTT (sec)	230 (3%)	35 [29–44]	33 [28–42]	38 [32–52]	0.001
Fibrinogen (mg/dL)	200 (16%)	360 [256–484]	373 [277–501]	311 [237–450]	0.023

*According to sepsis-3 criteria: Sepsis is defined as SOFA score increase ≥ 2 points and suspected infection by the treating physician. Septic shock is defined as sepsis plus vasopressor therapy to maintain mean arterial pressure ≥ 65 mmHg in the absence of hypovolemia and a lactate level > 2 mmol/L.

BMI = body mass index; SOFA = sequential organ failure assessment; AKI = acute kidney injury; suPAR = soluble urokinase plasminogen activator receptor; ICU = intensive care unit; CKD = chronic kidney disease; COPD = chronic obstructive pulmonary disease; HDL = high-density lipoprotein; INR = international normalized ratio; aPTT = activated partial thromboplastin time.

^#^AKI 3 criteria were fulfilled as following: creatinine levels (31%), reduced urinary output (19%), both (40%), initiation of RRT (10%).

### suPAR levels in the study population and subgroups

The median baseline suPAR level of the total cohort was 8.0 ng/mL [4.3–14.4, range: 1.5–56.8], with lower levels in ICU survivors than ICU non-survivors (6.7 vs. 11.6 ng/mL, p = 0.0003; Table 1). suPAR levels were significantly lower levels in patients with acute myocardial infarction and intoxicated patients, and significantly higher levels in COVID-19 patients, CKD patients, patients with moderate-severe liver disease, and patients with sepsis including those with septic shock (Table 2). Levels of suPAR were also higher in patients with AKI-3 on admission compared to those without AKI-3 (11.2 vs 6.6 ng/mL; p = 0.0001).

**Table 2:** suPAR in prespecified and exploratory subgroups.

	suPAR (ng/mL) Median [25th-75th percentile]		p
	No	Yes	
**Pre-specified subgroups**			
Active hematological malignant disease	8.3 [4.3–14.4]	6.2 [5.2–18.6]	0.945
Active cancer (non-hematological)	8.0 [4.3–14.4]	N/A	N/A
Moderate to severe liver disease	7.3 [4.0–13.6]	19.4 [11.7–34.9]	< 0.0001
Chronic kidney disease G3-5	5.9 [3.6–13.6]	11.1 [7.6–18.2]	< 0.0001
COPD GOLD 2–4 (pre-existing)	8.0 [4.1–14.5]	8.5 [5.3–13.6]	0.407
Acute myocardial infarction	8.5 [4.5–15.2]	3.5 [2.4–4.8]	0.0003
Elective valve surgery or PCI	8.2 [4.3–14.5]	5.2 [5.2–5.2]	0.574
Continuous RRT*	13.4 [7.1–19.8]	11.8 [8.3–25.4]	0.452
**Exploratory subgroups**			
Acute kidney injury on admission	6.6 [3.8–14.2]	11.2 [8.2–16.2]	0.0001
Neurological disease	8.3 [4.3–14.5]	7.5 [4.0–12.2]	0.797
Cardiopulmonary resuscitation	8.5 [4.3–15.2]	5.7 [3.9–7.8]	0.080
Hemorrhagic shock / GI bleeding	8.2 [4.3–14.4]	6.5 [4.2–15.2]	0.978
Intoxication	9.1 [4.5–15.2]	4.0 [2.9–5.3]	0.0003
COVID-19	7.4 [4.2–14.4]	11.7 [9.0–17.3]	0.009
Sepsis	4.6 [3.4–8.8]	13.0 [7.5–21.0]	< 0.0001
Septic shock	7.2 [4.0–13.6]	13.8 [7.5–23.2]	< 0.0001

Note that a patient may be in more than one group. * Out of 55 patients who received dialysis.

suPAR = soluble urokinase plasminogen activator receptor; N/A = not applicable; COPD = chronic obstructive pulmonary disease; GOLD = global initiative for chronic obstructive lung disease; PCI = percutaneous intervention; RRT = renal replacement therapy; GI = gastrointestinal; COVID-19 = coronavirus disease 2019.

suPAR levels positively correlated with SOFA score (rho = 0.47, p < 0.0001) and with the hemostasis parameters INR (rho = 0.36; p < 0.0001), aPTT (rho = 0.42; p < 0.0001), and fibrinogen (rho = 0.18; p = 0.012). CRP (rho = 0.50; p < 0.0001), PCT (rho = 0.62; p < 0.0001), and IL-6 (rho = 0.38; p < 0.0001) correlated significantly positively with suPAR levels, while white blood cell count (rho = 0.03; p = 0.607) did not.

### Univariable logistic regression analyses – ICU mortality

**Figure 1:**
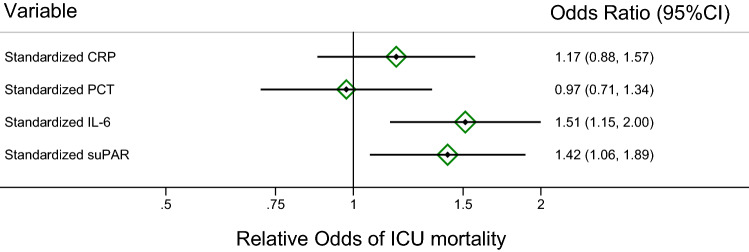
Z-standardized biomarkers for prediction of ICU mortality. z-standardization = all four biomarkers standardized to a mean of zero and a standard deviation (SD) of 1. Note that the hollow green diamonds represent the odds ratio for the respective biomarker, and the associated bars represented the 95% confidence interval. The black vertical line represents an odds ratio of 1 (“line of unity”). ICU = intensive care unit; CI = confidence interval; suPAR = soluble urokinase plasminogen activator receptor; CRP = C-reactive protein; PCT = procalcitonin; IL-6 = interleukin-6.

**Figure 2:**
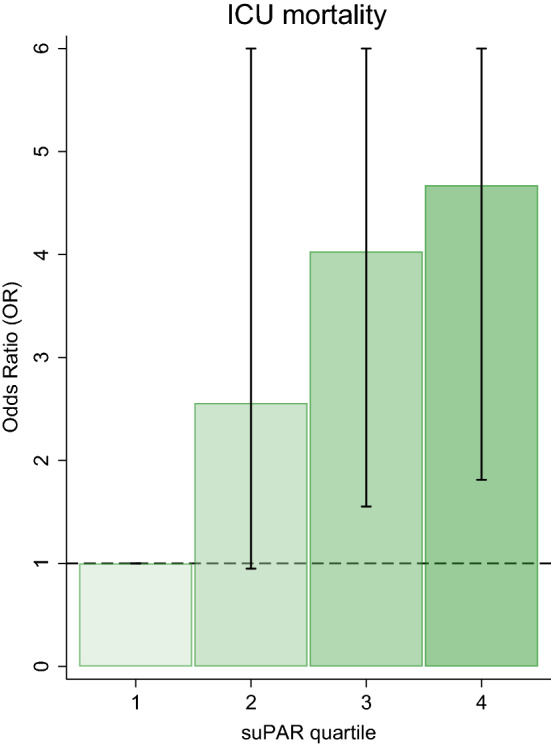
Association of ICU mortality with suPAR quartiles. The highest compared to the lowest quartile of suPAR had an odds ratio (OR) of 4.7 for ICU mortality. In the figure, the upper border of the confidence interval was truncated at the OR of 6.

In univariable logistic regression analyses treating ICU mortality as a binary outcome variable, higher suPAR, higher lactate, and higher SOFA were among others prognostic for higher risk of ICU mortality (Table 3). Next, we used z-standardization to compare the prognostic impact of CRP, PCT, IL-6, and suPAR on ICU mortality on a common scale (all four biomarkers standardized to mean of zero and standard deviation (SD) of 1). Only suPAR and IL-6 were significant predictors for ICU mortality, with 1SD increase in suPAR being associated with a 1.4-fold increase in the odds of ICU mortality, while Z-standardized CRP and PCT were not significantly associated with this endpoint (Table 3, Fig. 1). Furthermore, the highest compared to the lowest quartile of suPAR had an OR of 4.7 for ICU mortality (Fig. 2). The results for suPAR towards ICU mortality prediction were furthermore consistent among all pre-specified (Supplementary Fig. 2A) and exploratory (Supplementary Fig. 2B) subgroups.

**Table 3:** Univariable logistic regression analysis for ICU and 28-day mortality.

Variables	ICU mortality			28-day mortality		
	Odds Ratio (OR)	95% CI	p	Odds Ratio (OR)	95% CI	p
Age (per 5 years increase)	1.02	0.93–1.12	0.645	1.06	0.97–1.16	0.170
Female sex	0.81	0.45–1.48	0.499	0.86	0.50–1.47	0.571
suPAR (per 1 ng/mL increase)	1.04	1.01–1.06	0.011	1.03	1.00–1.06	0.038
suPAR (per doubling)	1.58	1.22–2.04	0.001	1.47	1.16–1.85	0.001
SOFA (per 1 point increase)	1.38	1.26–1.52	< 0.0001	1.29	1.19–1.39	< 0.0001
WBC (per 1 G/L increase)	1.05	1.01–1.09	0.016	1.04	1.00–1.08	0.033
Hb (per 1 g/dL increase)	0.94	0.84–1.04	0.228	0.93	0.84–1.02	0.131
CRP (per 10 mg/L increase)	1.01	0.99–1.04	0.323	1.01	0.99–1.04	0.264
CRP (per doubling)	1.06	0.94–1.18	0.342	1.08	0.97–1.19	0.157
PCT (per doubling)	1.12	1.02–1.24	0.023	1.08	0.98–1.18	0.109
IL-6 (per 100 pg/mL increase)	1.03	1.01–1.05	0.006	1.02	1.00–1.04	0.086
Albumin (per 1 g/dL increase)	0.51	0.32–0.80	0.003	0.45	0.30–0.69	< 0.0001
INR (per 1 unit increase)	1.43	1.07–1.91	0.017	1.27	0.96–1.68	0.092
aPTT (per 5 s increase)	1.04	0.98–1.10	0.167	1.02	0.97–1.07	0.492
Fib (per 100 mg/dL increase)	0.79	0.63–1.00	0.045	0.80	0.66–0.98	0.030
Chol (per 10 mg/dL increase)	0.95	0.89–1.02	0.131	0.98	0.96–0.99	0.509
HDL-C (per 10 mg/dL increase)	0.77	0.62–0.95	0.013	0.77	0.64–0.92	0.005
TG (per 10 mg/dL increase)	1.01	0.99–1.04	0.341	1.02	0.99–1.04	0.208
Lactate (per 1 mmol/L increase)	1.20	1.09–1.33	< 0.0001	1.14	1.04–1.25	0.006
Standardized biomarkers*						
CRP (per 1 SD increase)	1.17	0.88–1.57	0.287	1.20	0.91–1.57	0.196
PCT (per 1 SD increase)	0.97	0.71–1.34	0.873	0.85	0.61–1.20	0.360
IL-6 (per 1 SD increase)	1.51	1.15–2.00	0.004	1.30	0.99–1.70	0.063
suPAR (per 1 SD increase)	1.42	1.06–1.89	0.017	1.30	0.99–1.71	0.062

*Standardization performed for those n = 215 patients who had all 4 biomarkers observed.

Abbreviations: ICU = intensive care unit; CI = confidence interval; suPAR = soluble urokinase plasminogen activator receptor; SOFA = sequential organ failure assessment; WBC = white blood count; Hb = hemoglobin; CRP = C-reactive protein; PCT = procalcitonin; IL-6 = interleukin-6; INR = international normalized ratio; aPTT = activated partial thromboplastin time; Fib = fibrinogen; Chol = Cholesterol; HDL-C = high-density lipoprotein cholesterol; TG = triglycerides; SD = standard deviation.

### Multivariable analysis for ICU mortality

In multivariable logistic regression analysis, a stepwise-backwards selection algorithm with p > 0.10 as the criterion for removal resulted in a multivariable model with higher SOFA score and higher lactate as independent predictors of increased ICU mortality, and higher SOFA score as independent predictor of increased 28-day mortality, respectively (Table 4).

**Table 4:** Multivariable logistic regression analysis for ICU and 28-day mortality.

Model	Variable	Odds Ratio (OR)	95% confidence interval	p
Multivariable Model #1 – ICU mortality*	Lactate (per 1 mmol/L increase)	1.20	1.05–1.36	0.006
	SOFA score (per 1 point increase)	1.36	1.21–1.52	< 0.0001
Multivariable Model #2 – 28-day mortality**	Fibrinogen (per 100 mg/dL increase)	0.80	0.63–1.00	0.054
	SOFA score (per 1 point increase)	1.24	1.14–1.35	< 0.0001

*Variables included in backward selection process: soluble urokinase plasminogen activator receptor (suPAR) per doubling, procalcitonin, sequential organ failure assessment (SOFA) score, white blood count, international normalized ratio (INR), albumin, interleukin-6, lactate, high-density lipoprotein cholesterol (HDL-C), fibrinogen.

**Variables included in backward selection process: suPAR per doubling, SOFA score, white blood count, albumin, lactate, HDL-C, fibrinogen.

### Exploratory analysis of overall survival and suPAR levels

**Figure 3:**
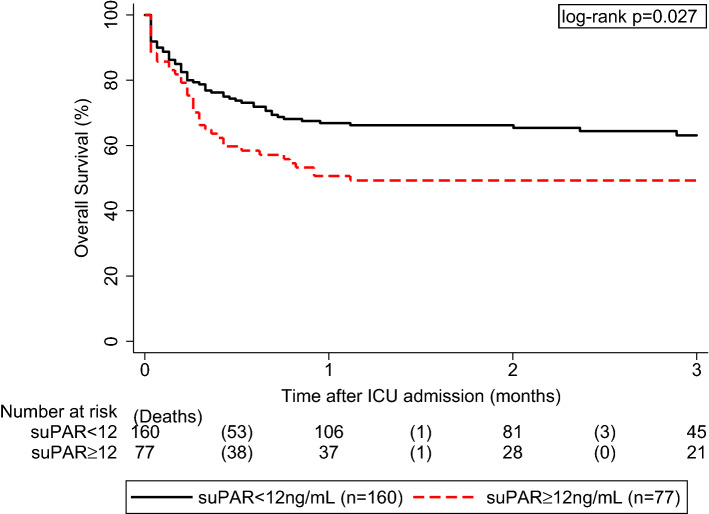
Overall survival for suPAR levels above or below a 12 ng/mL cutoff. Median follow-up of the total cohort was 2.9 months with 96 observed deaths from any cause. Using a previously published suPAR cut-off at 12 ng/mL for validation, Kaplan–Meier overall survival estimates at 3 months were 63% in the 160 patients with baseline suPAR levels below 12 ng/mL, and 49% in the 77 patients with baseline suPAR levels ≥ this cut-off (log-rank p = 0.027).

We observed 96 deaths (41%) from any cause during a median follow-up of 2.9 months. Using and validating a published suPAR cut-off at 12 ng/mL by Giamarellos-Bourboulis et al.^21^, Kaplan–Meier overall survival estimates at 3 months were 63% in the 160 patients with baseline suPAR levels below 12 ng/mL, and 49% in the 77 patients with baseline suPAR levels ≥ this cut-off (log-rank p = 0.027, Fig. 3).

### Mortality risk stratification in the ICU

**Figure 4:**
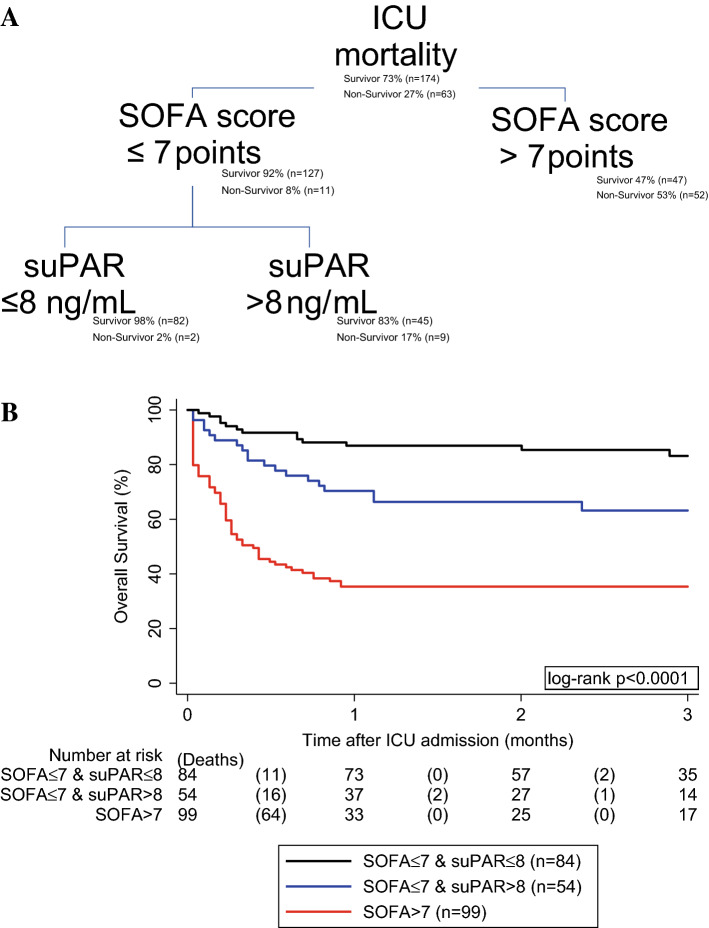
(**A**) Random forest analysis—overall survival according to risk stratification groups. For this analysis, we used the random forest machine learning method with the CHAID algorithm treating ICU mortality as a dependent variable and suPAR and SOFA score as independent variables, with tenfold cross validation. The random forest proposed cut-off values for SOFA score at 7 points (corrected p < 0.0001) and for suPAR at 8 ng/mL (corrected p = 0.022). Note that in patients with a SOFA score > 7 the prognosis was determined solely by the SOFA score. (**B**) Random forest analysis – Overall survival according to risk stratification groups. ICU mortality was 53% in patients with a SOFA score > 7 points (n = 99), 17% in patients with a SOFA score ≤ 7 points and suPAR levels > 8 ng/mL (n = 54), and 2% in patients with a SOFA score ≤ 7 points and suPAR levels ≤ 8 ng/mL (n = 84). Log-rank p < 0.0001.

We next assessed whether suPAR could improve mortality risk stratification towards ICU mortality beyond the SOFA score. In detail, the association between suPAR and higher risk of ICU mortality did not prevail after adjusting for SOFA score (adjusted OR for ICU mortality per doubling of suPAR = 1.08, 95% CI: 0.79–1.47, p = 0.636). However, we observed some evidence for an interaction between suPAR and SOFA (interaction p = 0.053), with higher SOFA scores reducing the prognostic impact of suPAR on ICU mortality, and vice versa (Supplementary Table 1). We thus employed a random forest to our ICU mortality data, which suggested SOFA score and suPAR cut-offs at 7 points and 8 ng/mL for ICU mortality risk stratification, respectively. In detail, the random forest suggested that prognosis is determined by the SOFA score in patients with > 7 points. However, in patients with a SOFA score ≤ 7 points, further prognosis is determined by suPAR levels at a cut-off at 8 ng/mL (Fig. 4A). Consequently, ICU mortality was 53% in the 99 patients with a SOFA score > 7 points, 17% in the 54 patients with a SOFA score ≤ 7 points and suPAR levels > 8 ng/mL, and 2% in the 84 patients with a SOFA score ≤ 7 points and suPAR levels ≤ 8 ng/mL. Furthermore, this association consists regarding overall survival (Fig. 4B, supplementary Fig. 3).

### suPAR prognostic performance regarding other clinical endpoints

Apart from mortality endpoints, we investigated other pre-specified and exploratory clinically relevant endpoints. In 12 patients, dialysis was performed before first sample of suPAR was drawn (median 7 h later), but there was no significant difference between suPAR levels before and after dialysis initiation (p = 0.959). We excluded patients with AKI-3 already being present on admission and investigated whether suPAR was able to predict the occurrence of AKI-3 during ICU stay. suPAR was a significant predictor for the occurrence of AKI-3 during ICU stay (suPAR per doubling OR 1.89, 95% CI: 1.20–2.98; p = 0.006). Furthermore, in the overall cohort, suPAR was, among others, also a predictor for the occurrence of the necessity of increasing catecholamine doses within the first 12 h after ICU admission (suPAR per doubling OR 1.48, 95% CI: 1.16–1.89; p = 0.002; Supplementary Table 2).

### suPAR levels at hospital discharge

Fifty-four patients had suPAR samples obtained within 72 h before alive hospital discharge, either directly from ICU or from general ward. The median suPAR concentration on discharge from hospital was 5.8 ng/mL [3.4–10.3]. The values were higher in patients with (8.4 ng/mL [4.8–14.5]) compared to those without unplanned 90-day readmission (5.2 ng/mL [3.3–8.5], p = 0.084). The suPAR levels at hospital discharge showed a borderline significant association with the rate of unplanned 90-day readmission in our cohort (OR per 1 ng/mL suPAR increase 1.12 [1.00–1.24], p = 0.053). This fact might be driven by the small sample size of this subgroup and needs additional investigation.

## Discussion

This study assessed suPAR levels on ICU admission in critically ill patients in a real-world setting. Levels of suPAR were significantly higher in ICU non-survivors compared to ICU survivors. Moreover, suPAR had the potential to further stratify and predict outcome in patients with moderate risk for ICU mortality according to SOFA score.

suPAR is a circulating protein and blood levels are low during physiological conditions^6^. suPAR is a sensitive but non-specific marker of acute disease, representing inflammation, the immune and coagulation system, and is also found on endothelial cells^5,7^. suPAR is not solely a representation of infection, but also mirrors comorbidities and organ dysfunctions^22^. suPAR originating from immature myeloid cells is furthermore part of the three-finger fold protein toxin superfamily with strong signaling capabilities^23–27^. It may come in different proteolytic fragments and isoforms, and can bind to integrins^6,16^. CRP, on the other hand, is a pentameric protein mainly synthesized in the liver with very low signaling properties^28^. Similarly, increased suPAR levels can be found in bronchoalveolar fluid during acute respiratory distress syndrome likely due to neutrophils present in lung tissue^29^. The normal blood levels for healthy individuals are reported to be 2–3 ng/ml^9,30^, and are clearly higher in ICU patients compared to patients outside the ICU^15^. In our study investigating critically ill ICU patients, suPAR levels were elevated at 8.0 [4.3–14.5] ng/mL. The previously reported mean difference of suPAR between sepsis survivors and non-survivors was 5.2 ng/ml^31^. We also found significantly lower suPAR levels in ICU survivors at 6.7 compared to non-survivors at 11.6 ng/mL. Furthermore, levels were elevated in patients with COVID-19 and sepsis likely due to the increased activity of immune cells. We can only speculate that the burden of inflammation and in parallel, the levels of suPAR are lower in patients with intoxication and acute myocardial infarction when compared to patients with sepsis^32,33^.

Higher suPAR levels have been associated with worse clinical outcome including higher 28-day mortality and rate of AKI in several selected patient cohorts^12,15,34–39^. In one study, suPAR was associated with clinical deterioration within 72 h in emergency department patients with bacterial sepsis^40^. In a study of ICU sepsis patients, suPAR results were also associated with hospital mortality but did not improve the area under the receiver operating characteristics (AUROC) of the SOFA or Acute Physiology And Chronic Health Evaluation (APACHE) score^41^. Also, the prognostic performance of suPAR was found to be worse than the Simplified Acute Physiology Score 2 (SAPS2) score^34^. A meta-analysis suggested the potential role of suPAR for the prediction of 28-day mortality in sepsis patients, but the reported AUROC (ranging from 0.670–0.788)^31^ were inferior to the SOFA score (0.773 [0.712–0.834]) and slightly higher than the AUROC of suPAR in our study (0.630 [0.559–0.702]). This difference may be explained by the fact that the aim of our study was to include a fairly heterogeneous (e.g., unselected) population of critically ill patients, while previous studies investigated a narrower patient cohort. Overall, we found that ICU non-survivors had higher levels of suPAR and that suPAR was a strong predictor of ICU mortality. Nevertheless, the prognostic value of the SOFA score for the overall cohort was not improved by adding suPAR to the model, and suPAR did not remain predictive in multivariable analysis. However, we found an interaction between the SOFA score and suPAR levels suggesting that the prognostic impact of suPAR decreased with increasing SOFA score. In the most severely ill patients with the highest rate of organ dysfunction, the prognostic performance of SOFA score outperformed suPAR and prognosis therefore was determined solely by the SOFA score. On the other hand, in moderately ill ICU patients as reflected by a SOFA score ≤ 7, suPAR was a strong marker to identify patients with an exceptionally good ICU prognosis independent of the underlying disease. Our findings are somewhat similar to another study that found that sepsis patients with an APACHE II score ≥ 17 and a suPAR level ≥ 12 ng/mL had the worst outcome, while patients with APACHE II < 17 and suPAR < 12 ng/mL had the best outcome^21^. We furthermore validated this previously reported cutoff of 12 ng/mL and found that overall survival estimates were significantly different between patients below or above this cutoff (63% vs. 49%).

The study by Hayek et al.also assessed critically ill patients and found that patients in the highest suPAR quartile had higher rates of AKI. In addition, in an animal model, the same group found that a monoclonal antibody therapy against suPAR attenuated the rate of AKI^17^. In another study, which involved adult hospitalized COVID-19 patients both inside and outside ICU, increasing suPAR tertiles were associated with AKI and dialysis^42^. Similarly, in our study, the rate of AKI-3 development during ICU stay in patients with AKI-3 not present on admission, was predicted by the suPAR level at the time of ICU admission. Therefore, future investigations should focus on suPAR as a potential therapeutic target to alter the clinical course on development of AKI-3. As an exploratory endpoint, we investigated whether suPAR levels on hospital discharge were able to predict unplanned 90-day readmission rate. Our data suggest that suPAR may be helpful in the decision process whether a patient can be safely discharged from the hospital; however, these results need to be validated in a separate larger trial.

Most previous studies used an ELISA to measure suPAR, which is time-consuming and often performed with some delay due to batch analyses. In our study, however, we used an automated immunoturbidometric assay, which can be run on routine laboratory analyzers and is therefore able to provide results within one hour. From this point of view, an implantation into clinical routine is more than promising. Intensive care scores, e.g. APACHE and SAPS, are time-consuming to calculate, and suPAR may therefore provide a quick stratification tool. Furthermore, suPAR outperformed other conventional inflammatory biomarkers such as CRP and PCT and was similar to IL-6 in prediction of ICU mortality in our cohort. Previous studies showed that suPAR levels remain stable over the first week^39,43,44^.

As a side note, like previous reports^45^, we were able to show that lipoproteins were significantly different between ICU survivors and non-survivors, with the latter having lower levels of HDL-C and total cholesterol. Most notably, HDL-C levels were significantly lower in patients with hemorrhagic shock or GI bleeding, moderate-severe liver disease and in sepsis patients (Supplementary Table 3).

Our study has several limitations. First, it was a single center study, therefore the external validity for other facilities may be reduced and results should be validated in larger multicentric trials. Second, our study was not designed for validation of an already approved turbidimetric immunoassay but to investigate its use in unselected critically ill patients for outcome prediction. However, based on our experience and the low workload for laboratory staff we assume that the turbidimetric test as used in our study provides much more clinical practicability. Third, currently there is no evidence that a prognostic biomarker improves outcomes even when the risk of a patient is clearly delineated. However, given the recent scientific data suPAR may not only have prognostic value but may also be a therapeutic target in the future. Fourth, our study was not powered for the 90-day readmission rate, so results must be interpreted as hypothesis-generating.

## Conclusion

In summary, suPAR was associated with ICU mortality in univariable logistic regression, and may offer the highest prognostic potential for risk stratification in moderately ill ICU patients as reflected by a SOFA score ≤ 7. Furthermore, our study shows that suPAR level on ICU admission may be a useful biomarker for prediction of AKI-3 in ICU patients.

## Supplementary Information

Supplementary Information.

## Data Availability

The datasets used and/or analysed during the current study are available from the corresponding author on reasonable request.
