# Supplementary material for: Soluble urokinase plasminogen activator receptor (suPAR) predicts critical illness and kidney failure in patients admitted to the intensive care unit
Source: Sci Rep. 2021 Sep 1;11:17476. doi: 10.1038/s41598-021-96352-1 (PMC8410930; doi:10.1038/s41598-021-96352-1)
Supplement: Supplementary file 1 — Supplementary Information. [file 41598_2021_96352_MOESM1_ESM.docx]

**Soluble urokinase plasminogen activator receptor (suPAR) predicts critical illness and kidney failure in patients admitted to the intensive care unit**

Alexander C. Reisinger, MD^1^ (ORCID ID: 0000-0002-5773-5695); Tobias Niedrist, MD²; Florian Posch, PhD^3^; Stefan Hatzl, MD^1,4^; Gerald Hackl, MD^1^; Juergen Prattes, MD^5^ (ORCID ID: 0000-0001-5751-9311); Gernot Schilcher, MD^1^; Anna-Maria Meißl, MD^6^; Reinhard B. Raggam, MD^7^; Markus Herrmann, MD^2^; Philipp Eller, MD^1*^

**Supplementary methods, figures and tables**

Supplementary Methods: Page 2

Supplementary Tables: Page 3-6

Supplementary Figures: Page 7-10

**Supplementary Methods:**

Blood cell count, plasma creatinine, bilirubin, albumin, C-reactive protein (CRP), procalcitonin (PCT), and interleukin-6 (IL-6), lipid and hemostasis parameters were measured using automated Sysmex (Sysmex Austria GmbH, Vienna, Austria), Cobas® (Roche Diagnostics, Mannheim, Germany) or Atellica COAG (Siemens Healthcare Diagnostics GmbH, Vienna, Austria) analyzers in the routine central laboratory unit. Laboratory value readings above the measurement range of the respective assay, i.e. international normalized ratio (INR) >8.9 (n=2 patients), activated partial thromboplastin time (aPTT) >160 seconds (n=5 patients), fibrinogen >900mg/dL (n=1 patient), and IL-6 >5000 pg/mL (n=13 patients), were truncated at the upper limit of the range of detection. For those patients who had values below the limit of detection, (CRP <0.6mg/L, n=5; PCT <0.02, n=1; IL-6 <1.5ng/mL, n=1), we simulated random values from a uniform distribution in the respective range. The daily coefficients of variation (CV) of the laboratory assay precision were smaller than 5.9 % in the study period.

Power calculation

We used results of suPAR which have been published before (Raggam *et al*., 2014): Levels of suPAR were 14.7 ng/mL (9.8-20) in non-survivors and 6.8 (4.7-10.2) in survivors. We considered a rate of death of 33% for the total ICU population. For a power regarding the primary endpoint of 0.90 at an alpha of 0.05, we estimated 40 patients. However, as the data of Raggam *et al* only included patients with SIRS and to account for the secondary endpoints, we chose a pragmatic time window of four month in this study to ensure sufficient power in the heterogenous intensive care population.

**Supplementary Table 1: Multivariable logistic regression model of suPAR and SOFA and their interaction towards ICU mortality.**

| **Variable** | **Odds Ratio (OR)** | **95%CI** | **p** |
| --- | --- | --- | --- |
|  |  |  |  |
| suPAR (per doubling) | 2.35 | 1.00-5.55 | 0.051 |
| SOFA score (per 1 point increase) | 1.83 | 1.33-2.52 | <0.0001 |
| suPAR ## SOFA score | 0.92 | 0.84-1.00 | 0.053 |

Higher SOFA score reduces the prognostic impact of suPAR on ICU mortality, and vice versa.

The interaction p-value was obtained by fitting a multivariable logistic regression model for ICU mortality with the three continuous explanatory variables SOFA score, suPAR, and their interaction. As usual for interaction analyses, we considered a "relaxed" alpha of 0.1 (i.e. p-values <0.10 were considered to indicate statistical significance).

Abbreviations: ICU = intensive care unit; CI = confidence interval; suPAR = soluble urokinase plasminogen activator receptor; SOFA = sequential organ failure assessment. ## denotes an interaction.

**Supplementary Table 2 Univariable logistic regression analyses for AKI-3 development during ICU stay and for increase in 12-hour vasopressor dose**

|  | **AKI-3 development during ICU stay  (n=15 out of 185 pts without AKI-3 on admission)** | | |  | **Increase in 12-hour vasopressor dose in  total cohort (n=237)** | | |
| --- | --- | --- | --- | --- | --- | --- | --- |
| **Variables** | **Odds Ratio (OR)** | **95%CI** | **p** |  | **Odds Ratio (OR)** | **95%CI** | **p** |
| Age (per 5 years increase) | 0.97 | 0.82-1.16 | 0.788 |  | 1.05 | 0.96-1.15 | 0.319 |
| Female sex | 1.59 | 0.55-4.60 | 0.389 |  | 1.08 | 0.62-1.88 | 0.790 |
| suPAR (per 1 ng/mL increase) | 1.05 | 1.01-1.09 | 0.016 |  | 1.03 | 1.01-1.06 | 0.016 |
| suPAR (per doubling) | 1.89 | 1.20-2.98 | 0.006 |  | 1.48 | 1.16-1.89 | 0.002 |
| SOFA (per 1 point increase) | 1.22 | 1.06-1.40 | 0.004 |  | 1.37 | 1.25-1.49 | <0.0001 |
| WBC (per 1 G/L increase) | 1.03 | 0.96-1.11 | 0.378 |  | 1.05 | 1.01-1.09 | 0.008 |
| Hb (per 1 g/dL increase) | 0.79 | 0.64-0.97 | 0.022 |  | 0.89 | 0.81-0.99 | 0.035 |
| CRP (per 10 mg/L increase) | 1.00 | 0.95-1.05 | 0.944 |  | 1.02 | 0.99-1.05 | 0.168 |
| CRP (per doubling) | 1.12 | 0.90-1.39 | 0.298 |  | 1.13 | 1.01-1.26 | 0.032 |
| PCT (per doubling) | 1.14 | 0.96-1.36 | 0.136 |  | 1.18 | 1.07-1.30 | 0.001 |
| IL-6 (per 100 pg/mL increase) | 1.01 | 0.97-1.05 | 0.689 |  | 1.03 | 1.00-1.05 | 0.016 |
| Albumin (per 1 g/dL increase) | 0.66 | 0.30-1.45 | 0.303 |  | 0.44 | 0.29-0.68 | <0.0001 |
| INR (per 1 unit increase) | 0.97 | 0.52-1.81 | 0.922 |  | 1.38 | 1.03-1.86 | 0.029 |
| aPTT (per 5 sec increase) | 1.02 | 0.94-1.11 | 0.590 |  | 1.04 | 0.99-1.10 | 0.113 |
| Fib (per 100 mg/dL increase) | 0.95 | 0.66-1.37 | 0.785 |  | 0.88 | 0.72-1.08 | 0.219 |
| Chol (per 10 mg/dL increase) | 1.04 | 0.97-1.13 | 0.281 |  | 0.91 | 0.84-0.97 | 0.005 |
| HDL-C (per 10 mg/dL increase) | 0.76 | 0.52-1.10 | 0.144 |  | 0.79 | 0.66-0.96 | 0.015 |
| TG (per 10 mg/dL increase) | 1.03 | 1.00-1.06 | 0.042 |  | 1.01 | 0.99-1.03 | 0.431 |
| Lactate (per 1 mmol/L increase) | 1.04 | 0.88-1.22 | 0.666 |  | 1.11 | 1.01-1.20 | 0.022 |

Abbreviations: ICU = intensive care unit; CI = confidence interval; suPAR = soluble urokinase plasminogen activator receptor; SOFA = sequential organ failure assessment; WBC = white blood count; Hb = hemoglobin; CRP = C-reactive protein; PCT = procalcitonin; IL-6 = interleukin-6; INR = international normalized ratio; aPTT = activated partial thromboplastin time; Fib = fibrinogen; Chol = Cholesterol; HDL-C = high-density lipoprotein cholesterol; TG = triglycerides; AKI-3 = acute kidney injury grade 3.

**Supplementary Table 3: Lipoproteins in prespecified and exploratory subgroups**

|  |  | **Cholesterol (mg/dL)** | | |  | **HDL cholesterol (mg/dL)** | | |  | **Triglycerides (mg/dL)** | | |
| --- | --- | --- | --- | --- | --- | --- | --- | --- | --- | --- | --- | --- |
| **Pre-specified subgroups** |  | **No** | **Yes** | **p** |  | **No** | **Yes** | **p** |  | **No** | **Yes** | **p** |
| Active hematological malignancy |  | 121 [93-157] | 113 [90-152] | 0.903 |  | 34 [22-45] | 22 [15-37] | 0.059 |  | 115 [85-165] | 191 [151-223] | 0.002 |
| Active cancer (non-hematological) |  | 120 [92-157] | N/A | N/A |  | 34 [22-45] | N/A | N/A |  | 120 [87-173] | N/A | N/A |
| Moderate to severe liver disease |  | 128 [97-164] | 83 [55-98] | <0.001 |  | 35 [25-46] | 14 [10-18] | <0.001 |  | 124 [94-176] | 96 [68-134] | 0.013 |
| Chronic kidney disease 3-5 |  | 126 [92-165] | 118 [98-142] | 0.542 |  | 34 [22-45] | 33 [23-42] | 0.753 |  | 110 [83-177] | 134 [105-169] | 0.068 |
| COPD GOLD 2-4 (pre-existing) |  | 121 [93-155] | 110 [78-169] | 0.311 |  | 33 [22-43] | 35 [21-52] | 0.348 |  | 122 [91-177] | 104 [71-160] | 0.113 |
| Acute myocardial infarction |  | 119 [92-152] | 159 [143-199] | 0.010 |  | 33 [21-43] | 43 [35-47] | 0.023 |  | 120 [87-174] | 103 [79-167] | 0.604 |
| Elective valve surgery or PCI |  | 119 [92-157] | 143 [143-143] | 0.591 |  | 34 [22-45] | 37 [37-37] | 0.782 |  | 120 [87-173] | 55 [55-55] | 0.140 |
| Continuous RRT* |  | 123 [109-146] | 116 [92-158] | 0.518 |  | 46 [38-52] | 30 [17-40] | 0.009 |  | 135 [124-184] | 135 [108-191] | 0.874 |
|  |  |  |  |  |  |  |  |  |  |  |  |  |
| **Exploratory subgroups** |  | **No** | **Yes** | **p** |  | **No** | **Yes** | **p** |  | **No** | **Yes** | **p** |
| Acute kidney injury on admission |  | 120 [93-155] | 124 [88-169] | 0.957 |  | 35 [23-46] | 29 [18-39] | 0.038 |  | 116 [85-168] | 131 [97-191] | 0.243 |
| Neurological disease |  | 119 [92-155] | 146 [121-188] | 0.216 |  | 34 [22-44] | 37 [18-52] | 0.882 |  | 120 [87-174] | 108 [92-159] | 0.696 |
| Cardiopulmonary resuscitation |  | 119 [92-155] | 140 [101-183] | 0.200 |  | 34 [21-44] | 38 [33-56] | 0.159 |  | 120 [87-172] | 107 [85-232] | 0.927 |
| Hemorrhagic shock / GI bleeding |  | 125 [94-161] | 78 [52-111] | <0.001 |  | 34 [23-46] | 14 [12-29] | <0.001 |  | 122 [92-174] | 85 [68-170] | 0.165 |
| Intoxication |  | 119 [92-155] | 156 [111-174] | 0.096 |  | 33 [21-43] | 44 [37-49] | 0.010 |  | 120 [87-170] | 150 [90-274] | 0.374 |
| COVID-19 |  | 122 [92-158] | 117 [85-154] | 0.641 |  | 34 [22-46] | 24 [22-34] | 0.097 |  | 120 [87-169] | 129 [95-192] | 0.665 |
| Sepsis |  | 137 [106-173] | 103 [86-138] | <0.001 |  | 39 [32-53] | 27 [16-34] | <0.001 |  | 107 [74-157] | 135 [103-191] | 0.002 |
| Septic shock |  | 128 [97-158] | 92 [83-134] | 0.009 |  | 35 [22-46] | 30 [14-38] | 0.020 |  | 121 [85-174] | 120 [91-170] | 0.874 |

Note that a patient may be in more than one group. * Out of 55 patients who received dialysis (47 values).

Abbreviations: N/A = not applicable; COPD = chronic obstructive pulmonary disease; GOLD = global initiative for chronic obstructive lung disease; PCI =percutaneous intervention; RRT = renal replacement therapy; GI = gastrointestinal; COVID-19 = coronavirus disease 2019.

**Supplementary Figure 1: Flowchart**


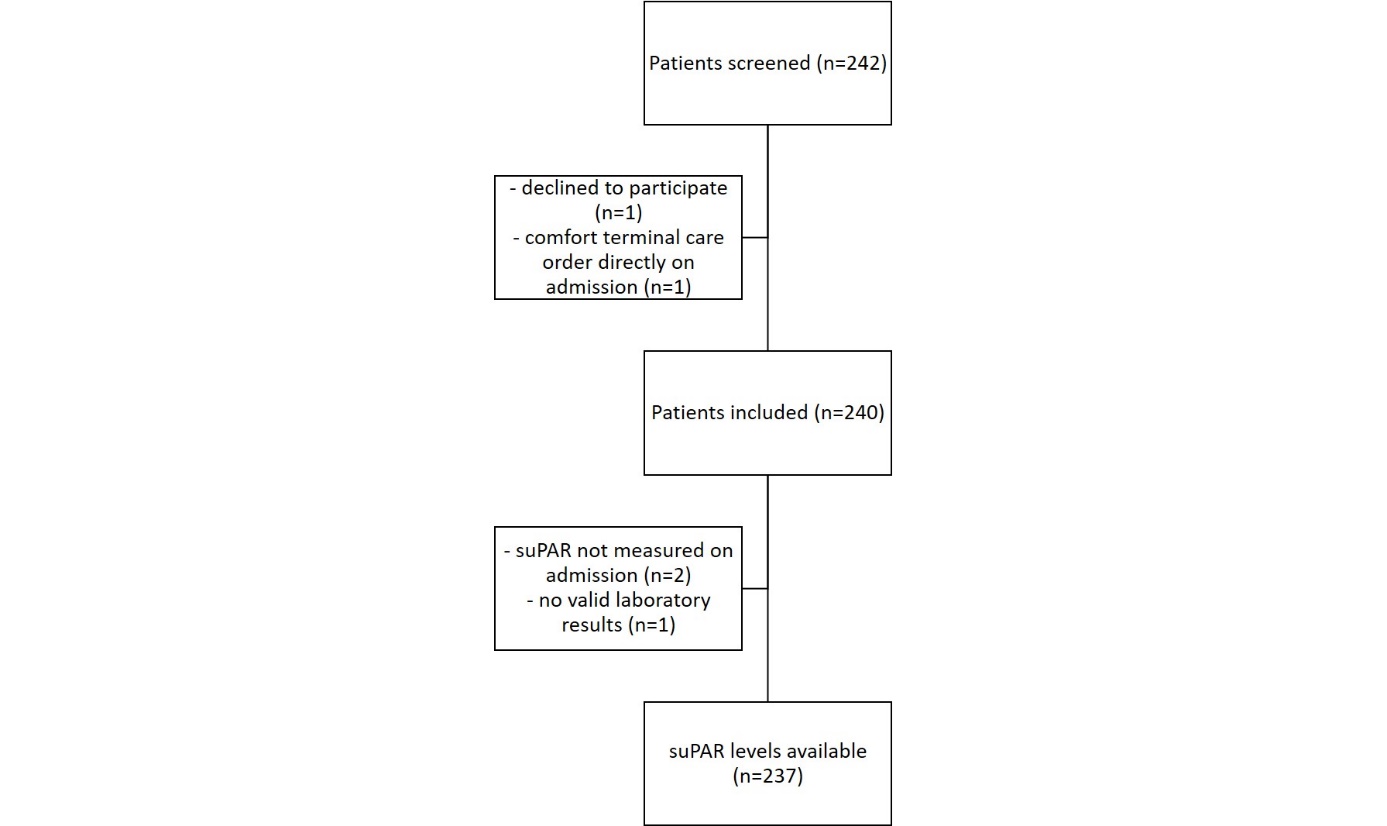


242 patients were initially considered for the study. 2 patients fulfilled exclusion criteria (1 patients declined to participate and 1 patient received a comfort terminal care order directly on admission). In two patients, suPAR measurements were not available on admission and one patient had invalid laboratory suPAR results despite several diluted and repeated measurements suggesting the presence of heterophilic antibodies. Therefore, in final analyses, 237 patients were included.

**Supplementary Figures 2A & 2B: Forest plot of the relative association of suPAR with ICU mortality in pre-specified and exploratory subgroups**

Note that the hollow green diamonds represent the odds ratio for ICU mortality, and the associated bars represented the 95% confidence interval. The black vertical line represents an odds ratio of 1 (“line of unity”). Regression was performed for the association of suPAR with ICU mortality in the pre-specified and exploratory subgroups.

**Supplementary Figure 3: Overall survival for suPAR levels above or below an 8 ng/mL cutoff**


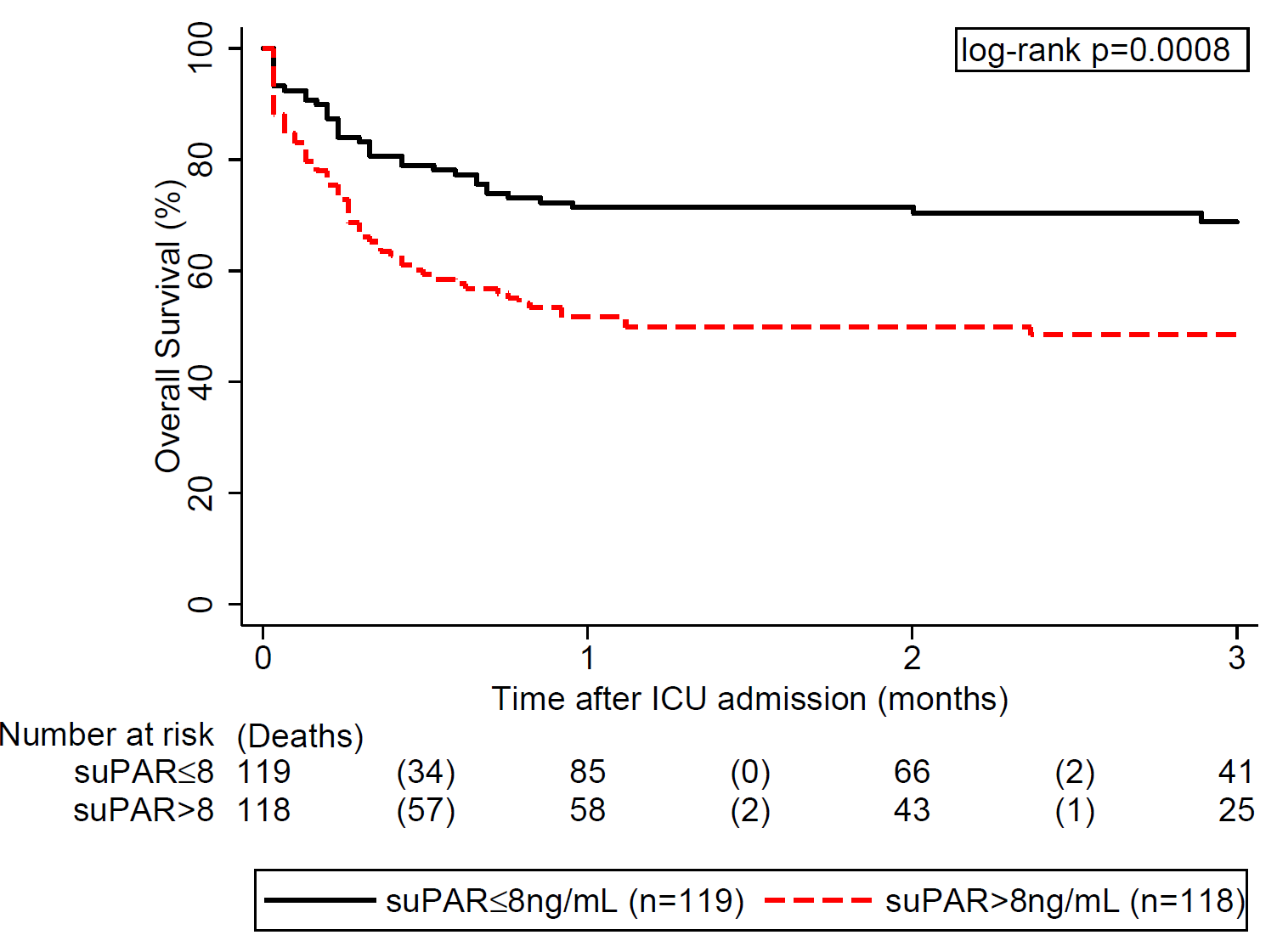


Kaplan-Meier plots of patients with suPAR ≤8 ng/mL (n=119) and suPAR >8ng/mL (n=118).
